# Evaluation of an Artificial Intelligence System for Detection of Invasive Lobular Carcinoma on Digital Mammography

**DOI:** 10.7759/cureus.38770

**Published:** 2023-05-09

**Authors:** Sylvia Arce, Arunima Vijay, Eunice Yim, Lisa R Spiguel, Mariam Hanna

**Affiliations:** 1 Department of Radiology, University of Florida College of Medicine, Gainesville, USA; 2 Department of Surgery, University of Florida College of Medicine, Gainesville, USA

**Keywords:** mammogram, convolutional neural networks (cnn), computer-aided diagnosis, breast cancer, artificial intelligence (ai), invasive lobular breast carcinoma

## Abstract

Introduction

Early breast cancer detection with screening mammography has been shown to reduce mortality and improve breast cancer survival. This study aims to evaluate the ability of an artificial intelligence computer-aided detection (AI CAD) system to detect biopsy-proven invasive lobular carcinoma (ILC) on digital mammography.

Methods

This retrospective study reviewed mammograms of patients who were diagnosed with biopsy-proved ILC between January 1, 2017, and January 1, 2022. All mammograms were analyzed using cmAssist^®^ (CureMetrix, San Diego, California, United States), which is an AI CAD for mammography. The AI CAD sensitivity for detecting ILC on mammography was calculated and further subdivided by lesion type, mass shape, and mass margins. To account for the within-subject correlation, generalized linear mixed models were implemented to investigate the association between age, family history, and breast density and whether the AI detected a false positive or true positive. Odds ratios, 95% confidence intervals, and p-values were also calculated.

Results

A total of 124 patients with 153 biopsy-proven ILC lesions were included. The AI CAD detected ILC on mammography with a sensitivity of 80%. The AI CAD had the highest sensitivity for detecting calcifications (100%), masses with irregular shape (82%), and masses with spiculated margins (86%). However, 88% of mammograms had at least one false positive mark with an average number of 3.9 false positive marks per mammogram.

Conclusion

The AI CAD system evaluated was successful in marking the malignancy in digital mammography. However, the numerous annotations confounded the ability to determine its overall accuracy and this reduces its potential use in real-life practice.

## Introduction

Breast cancer is the second leading cause of cancer death in women worldwide with over two million annual diagnoses [1]. Early breast cancer detection with screening mammography can reduce mortality by 20-35% and significantly improve breast cancer survival [2-4]. In the United States, almost 40 million mammographic exams are performed annually, most of which are done as part of screening programs [5]. This generates a large volume of mammograms that are manually interpreted by radiologists. However, manual analysis is both time and labor-intensive.

To assist radiologists and alleviate the problems associated with manual interpretation of mammograms, commercial computer-aided detection (CAD) systems for screening mammography were approved by the Food and Drug Administration in the 1990s [6]. Despite early promise, subsequent studies found that CAD did not improve the diagnostic accuracy of mammography [7-9]. A recent solution to improve diagnostic accuracy has been to integrate artificial intelligence into CAD software (AI CAD) [10]. The development of novel algorithms based on convolutional neural networks, commonly used for image recognition and classification, has allowed for significant advancements in AI CAD performance [10-11]. Research suggests that this new AI CAD may be used as a “pre-reader” or “second reader” for mammograms to ease the burden of manual interpretation [10,12].

Several studies have shown that AI CAD systems evaluating digital mammograms can achieve a breast cancer detection rate comparable to that of a radiologist [13-15]. However, these studies had some limitations. Most studies were based on cancer-enriched datasets rather than actual screening mammogram data [14]. Furthermore, the radiologists that the AI CAD system was compared with showed a poor performance [15]. Finally, AI CAD algorithms were often not publicly available, making it difficult to understand how the AI CAD arrived at the result [16]. Several AI CAD programs exist, but most have not been validated by an independent third-party [16]. 

To the best of our knowledge, there is currently no research regarding the application of AI CAD for detection of invasive lobular carcinoma (ILC) on mammography. The lack of work in this area is likely due to the limited number of ILC cases, with ILC representing 5-15% of new breast cancer diagnoses annually [17]. Mammography can detect ILC with a sensitivity of 57-81% [17]. This relatively low sensitivity is partly due to the histological pattern of ILC, which presents diffusely with a single-cell pattern of growth throughout the stroma, and without significant desmoplastic reaction [18]. If AI CAD could improve the detection of ILC on mammography, this may prevent the need for more costly and time-consuming imaging studies such as MRI.

This study evaluates the ability of a commercially available AI CAD to identify biopsy-proven ILC on digital mammography. This study also assesses what characteristics are associated with false positive rates when using the AI CAD.

## Materials and methods

The University of Florida (UF) Institutional Review Boards issued approval (IRB# 202200981) for this retrospective study to review mammograms of UF patients diagnosed with biopsy-proven ILC between January 1, 2017, and January 1, 2022. Patients aged 30 to 84 years old, with mammograms consisting of four standard views (left cranial-caudal, left mediolateral oblique, right cranial-caudal, right mediolateral oblique), and a pathology report confirming ILC were included. Patients were excluded if they lacked a proper mammogram, if there was an inability to access CAD data for their imaging, or if their pathology report was negative for ILC.

**Figure 1:**
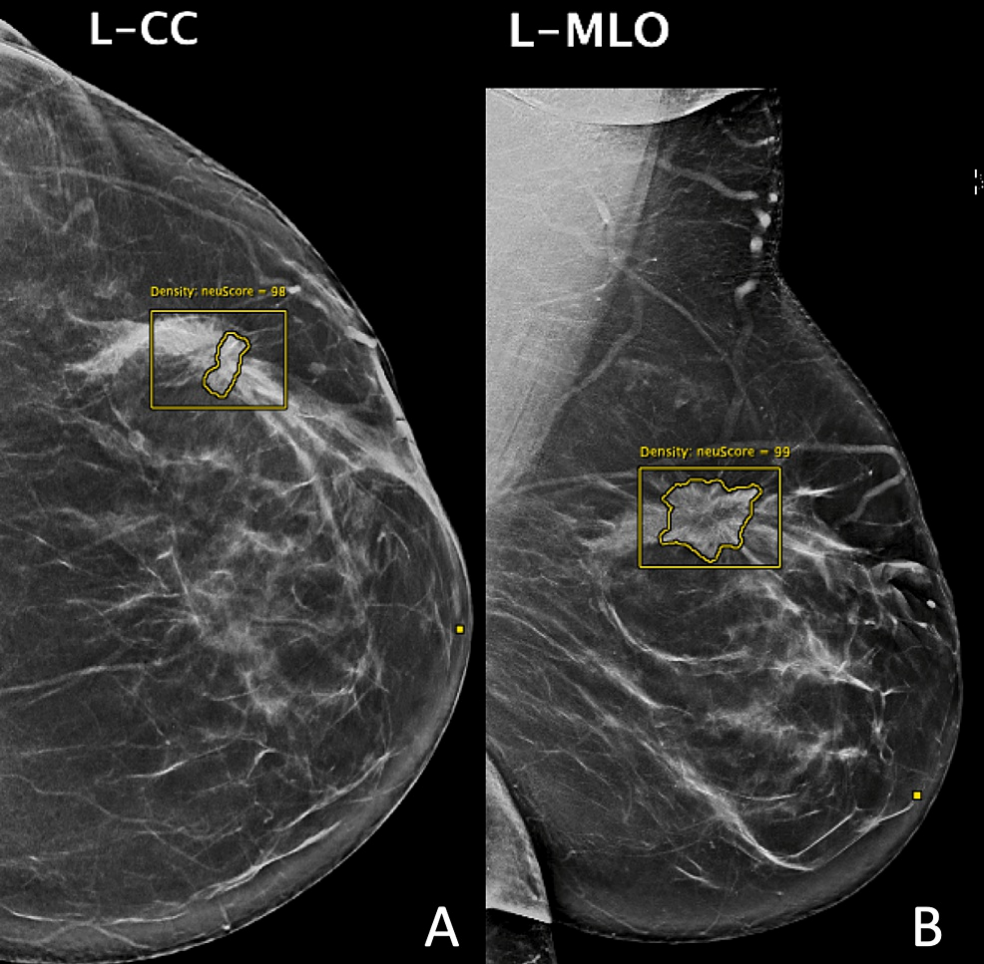
Example of cmAssist® AI CAD marking Mammogram of a 55-year-old woman with heterogeneously dense breasts. (A) Left cranial-caudal (L-CC) view and (B) left mediolateral oblique (L-MLO) view of invasive lobular carcinoma (ILC) presenting as a mass with spiculated margins extending over a 5 x 2 cm region in the left breast. The AI CAD detected the mass as a density and gave it a neuScore™of 99. Core needle biopsy revealed grade 2 ILC. cmAssist®: CureMetrix, San Diego, California, United States

The mammograms were analyzed using cmAssist® (CureMetrix, San Diego, California, United States), which is an AI CAD for mammography. cmAssist was trained using mammograms with biopsy-proven benign and malignant lesions and normal mammograms from multiple institutions [19]. cmAssist places markings on a mammogram, labels each marking as a “density” or a “calcification”, and assigns each marking a quantitative score (neuScore™) on a scale of 0-100, with higher numbers corresponding to a greater likelihood of malignancy. Figure 1 is an example of how the cmAssist AI CAD marks a spiculated mass in a patient with heterogeneously dense breasts. No mammograms analyzed in this study were used in the development of cmAssist.

Variables included in the dataset for analysis were age, family history of breast cancer (positive if the patient had two or more first-degree relatives with breast cancer, one first-degree relative with a *BRCA* mutation, or a personal history of a *BRCA* mutation), radiologic findings of biopsy-proven cancer (mass, calcification, asymmetry, or distortion), mass size (less than 1 cm or not), mass margins, breast density (fatty, scattered fibroglandular, heterogeneously dense, and extremely dense), mammogram findings and impression, Breast Imaging Reporting & Data System (BI-RADS®) category, ultrasound findings and impression, pathology findings and impression from the breast biopsy, and surgical pathology results. The number and type of marking made by cmAssist were recorded for each mammogram. cmAssist was considered to have correctly marked the lesion location if the AI CAD marking overlapped an area of concern identified in the radiology report. Additionally, cmAssist was considered to have correctly marked the lesion type if it labeled a finding as a “calcification” and the radiologist called it a calcification, or if it labeled a finding as a “density” and the radiologist called it a mass, asymmetry, or distortion. The ground truth was defined in terms of whether ILC was present or absent, and confirmed by histopathology from the breast biopsy.

The AI CAD sensitivity for detecting ILC on mammography was calculated and further subdivided by lesion type, mass shape, and mass margins. To account for the within-subject correlation, generalized linear mixed models were implemented to investigate the association between age, family history, and breast density and whether the AI detected a false positive or true positive. Odds ratios (ORs), 95% confidence intervals (CIs), and p-values were calculated and statistical significance was set at p < 0.05.

## Results

A total of 124 patients with biopsy-proven ILC were included in this study. Some patients had multiple ILC lesions in one or both breasts, so the total number of ILC lesions was 153. The AI CAD detected ILC on mammography with a sensitivity of 80% (95%CI: 0.73-0.86). The AI CAD had the highest sensitivity for detecting calcifications (100%; 95%CI: 0.87-1.00), and the lowest sensitivity for detecting distortions (55%; 95%CI: 0.33-0.75) (Table 1). Analysis of imaging characteristics showed that the AI CAD had the highest sensitivity for detecting masses with irregular shapes (82%; 95%CI: 0.60-0.95) (Table 2). It also had a sensitivity of 86% (95%CI: 0.71-0.95) for detecting masses with spiculated margins (Table 3). Eighty-eight percent of mammograms had at least one false positive mark with an average number of 3.9 false positive marks per mammogram. Patients who were older (OR=1.02; 95%CI: 1.00-1.04), had a positive family history (OR=1.8; 95%CI: 1.02-3.31), or had extremely dense breast density (OR=7.4; 95%CI: 2.00-28.56) were more likely to have a false positive mark detected by the AI CAD (all p < 0.05) (Table 4).

**Table 1:** Sensitivity of AI CAD detection on mammography by lesion type AI CAD: artificial intelligence computer-aided detection

Lesion Type	n = 153	Sensitivity (%)	95% Confidence Interval
Mass	74	80	0.68 – 0.89
Distortion	22	55	0.33 – 0.75
Asymmetry	23	78	0.56 – 0.93
Calcification	34	100	0.87 – 1.00

**Table 2:** Sensitivity of AI CAD detection on mammography by mass shape AI CAD: artificial intelligence computer-aided detection

Mass Shape	n = 24	Sensitivity (%)	95% Confidence Interval
Oval	1	0	0.00 – 0.97
Round	1	100	0.03 – 1.00
Irregular	22	82	0.60 – 0.95

**Table 3:** Sensitivity of AI CAD detection on mammography by mass margins AI CAD: artificial intelligence computer-aided detection

Mass Margins	n = 53	Sensitivity (%)	95% Confidence Interval
Spiculated	37	86	0.71 – 0.95
Obscured	8	88	0.47 – 1.00
Indistinct	5	60	0.15 – 0.95
Circumscribed	3	67	0.09 – 0.99

**Table 4:** Generalized linear mixed model for false positives of AI CAD detection AI CAD: artificial intelligence computer-aided detection

	Odds Ratio	95% Confidence Interval	p-value
Intercept	0.37	0.07 - 1.78	0.2089
Age (years)	1.02	1.00 - 1.04	0.0306
Family History of Breast Cancer	1.80	1.02 - 3.31	0.0479
Scattered vs Fatty	1.90	0.74 - 4.72	0.1692
Heterogeneously dense vs Fatty	1.71	0.66 - 4.24	0.2485
Extremely dense vs Fatty	7.38	2.00 - 28.56	0.0027

## Discussion

This study demonstrates that recent advances in AI algorithms have narrowed the gap between computer systems and human experts for detecting breast cancer in digital mammography. The sensitivity of the AI CAD for detecting ILC on mammography was 80%, which is comparable to the sensitivity of radiologists (between 57% and 81%) [17]. ILC most commonly presents as a spiculated, ill-defined mass or as an architectural distortion on mammography [17]. The AI CAD was successful at detecting masses with spiculated margins, suggesting that it is well suited to detect the subtle features of ILC in mammography. Many countries engage in double reading of mammograms to improve the sensitivity of detecting cancer [20]. Given the AI CAD’s sensitivity for ILC detection, it is possible that this technology could be used as a second reader or as part of a radiologist’s toolkit.

Despite its promise, AI CAD still has room for improvement. With a false positive rate of 3.9 marks per mammogram, radiologists using cmAssist must be able to identify and dismiss false positives to prevent an increase in screening recall rates. Also, this could increase the reading time of radiologists significantly. False positives are associated with patient anxiety, benign biopsies, unnecessary intervention or treatment, and increased healthcare spending [21]. The annual cost of false-positive mammograms and overdiagnosis of breast cancer is $4 billion among women aged 40-59 years in the United States [22]. Given that two-dimensional (2D) mammographic screening is the most commonly used screening method worldwide, the accuracy of mammographic readings must be preserved [23].

The limitations of this study include its retrospective design, single institution dataset, and the fact that the dataset only contains biopsy-proven cancer. Because all patients in this dataset had cancer, the specificity of the AI CAD could not be calculated, and a receiver operating characteristic analysis could not be performed. Since AI CAD is intended for use in a screening setting, the performance of AI CAD systems should also be tested on a screening population that includes normal mammograms.

Future work should focus on external validation studies that demonstrate AI CAD’s performance in a screening setting using a population with diverse racial and ethnic backgrounds, breast cancer risk factors, imaging vendors, and imaging modalities (e.g. digital breast tomosynthesis vs digital mammography). This data could then be compared to the radiologists’ performance in that setting. A prospective, randomized controlled trial involving multiple institutions is needed to further validate the AI CAD system.

## Conclusions

The AI CAD system evaluated was successful in marking malignancies although numerous annotations confounded the ability to determine its overall accuracy and decreases its use in real-life practice. However, with improvement in technology, AI CAD can have important implications for screening mammography programs that employ double reading, which is more costly and labor-intensive than single readings. Adoption of the AI CAD as a second reader could significantly decrease the workload of radiologists. Although promising, the AI CAD system can be improved to minimize the number of false positive markings and requires further validation in a screening setting.
